# Epidemiology of reported *Yersinia enterocolitica *infections in Germany, 2001-2008

**DOI:** 10.1186/1471-2458-10-337

**Published:** 2010-06-14

**Authors:** Bettina M Rosner, Klaus Stark, Dirk Werber

**Affiliations:** 1 Robert Koch Institute, Department of Infectious Disease Epidemiology, DGZ-Ring 1, 13086 Berlin, Germany

## Abstract

**Background:**

Yersiniosis is the third most common zoonotic bacterial disease in Germany and the European Union. Sequelae of *Yersinia enterocolitica *infections, such as reactive arthritis, have been reported. Consumption of pork and its products, especially eaten raw or undercooked, is an important risk factor of yersiniosis. Infection with *Y. enterocolitica *is notifiable through the national surveillance system for infectious diseases in Germany and several thousands of cases are being reported each year. We present recent data on the epidemiology of reported yersiniosis in Germany.

**Methods:**

Surveillance data on yersiniosis, accessed through the national level database (SurvNet), were analyzed with regard to time trends, demographical and geographical distribution, serotypes, and hospitalization, for the time period 2001-2008.

**Results:**

A total of 47,627 cases of yersiniosis were reported. The mean annual incidence of yersiniosis was 7.2/100,000 population. A downward trend in the number of reportable cases has occurred since 2002. Almost all *Y. enterocolitica *infections were reported as single cases, i.e., with no apparent links to other cases. The number of reported infections showed substantially less seasonal variation than in other zoonotic enteric diseases. The incidence was highest in children under five years (58/100,000 population), in particular in one-year-old children (108/100,000 population). Almost 97% of infections were acquired domestically. High incidences occurred in the eastern German federal states Thuringia, Saxony, and Saxony-Anhalt. Differences in incidences across federal states were driven primarily by incidence differences in children under five years. Hospitalization was reported for 17% of cases, the proportion being highest among teenagers. Almost 90% of *Y. enterocolitica *strains were diagnosed as serotype O:3, which is the serotype most frequently isolated from pigs.

**Conclusions:**

Yersiniosis is a zoonotic foodborne disease of relevance to public health in Germany because of its high incidence and risk for sequelae. The incidence of reported yersiniosis in Germany varies markedly from state to state, mainly due to incidence difference among young children. More research efforts should be directed towards the elucidation of risk factors of yersiniosis in this age group.

## Background

Yersiniosis due to infection with the bacterium *Yersinia enterocolitica *is a zoonotic gastrointestinal disease in humans. *Y. enterocolitica *species can be isolated from a variety of domestic and wildlife animals, e.g., pigs, cattle, sheep, goats, dogs, cats, wild boars, and small rodents [1]. Pigs are considered to be the main reservoir of human pathogenic strains, largely because of the high prevalence of these strains in pigs and the high genetic similarity between porcine and human isolates [2-4]. Infections are thought to be primarily transmitted to humans by food, in particular, raw or undercooked pork and pork products [1,5]. However, other risk factors, such as contaminated drinking water or pet animal contact, have been reported [6-9]. Six different biotypes (biotype 1A, 1B, 2-5) and numerous serotypes of *Y. enterocolitica *have been described. Eleven of those serotypes have frequently been associated with infections in humans [1]. In Europe, most of the human pathogenic *Y. enterocolitica *strains are classified as biotype 4, serotype O:3 [10]. Clinical symptoms of yersiniosis first appear after an incubation period of about 5 days (range 1-11 days) and include diarrhea, fever, vomiting, tenesma and abdominal pain. In older children and young adults, abdominal pain in the right lower abdomen can occur, which may be mistaken for appendicitis (pseudoappendicitis). Typically, symptoms disappear within 1-2 weeks after onset. Sequelae such as reactive arthritis or erythema nodosum sometimes occur [11]. Yersiniosis contributes substantially to foodborne diseases in industrialized countries and is therefore notifiable through national surveillance systems in most countries within the European Union (EU), including Germany. After campylobacteriosis and salmonellosis, yersiniosis ranks third among the notifiable bacterial zoonoses in Germany and the EU. In 2007, 8,874 confirmed cases of yersiniosis were reported to the European Centre for Disease Control and Prevention (ECDC), 4,987 (56%) of which were from Germany [12]. There is a paucity of recent comprehensive data on the epidemiology of yersiniosis, e.g., detailed analysis of trends over time or affected population groups.

## Methods

Yersiniosis surveillance data from Germany were analyzed for the time period 2001-2008. An acute culture-confirmed infection with *Yersinia enterocolitica *is notifiable to the local health department according to the Protection against Infection Act (Infektionsschutzgesetz, IfSG) of 2001. Each notification has to be electronically forwarded from the local health department via the state health department to the federal public health institute, the Robert Koch Institute (RKI), where the national surveillance database is hosted. To ensure comparability of surveillance data across federal states, surveillance case definitions exist for each notifiable condition. A case of yersiniosis is included in the RKI statistics when the diseased person being reported as a case showed clinical symptoms (i.e. at least one of the following: diarrhea, abdominal pain, tenesma, fever with body temperature of 38.5°C or above, and vomiting) and the *Y. enterocolitica *infection was either culture-confirmed from stool or some other clinical material, or confirmed epidemiologically. Epidemiological confirmation of a case is defined as contact with another laboratory-confirmed case, contact with an animal infected with *Y. enterocolitica*, or consumption of food items contaminated with *Y. enterocolitica*. Prior to 2004, patients with clinical symptoms and serological evidence of infection (agglutination reaction (Widal), confirmation of IgA-, IgG- or IgM-antibodies by ELISA or Western blot) also fulfilled the case definition. Data were accessed through the national level database (SurvNet) at the RKI and analyzed with Microsoft Excel. Data are openly available via SurvStat@RKI http://www3.rki.de/SurvStat/[13,14].

## Results

### Time trend

**Figure 1:**
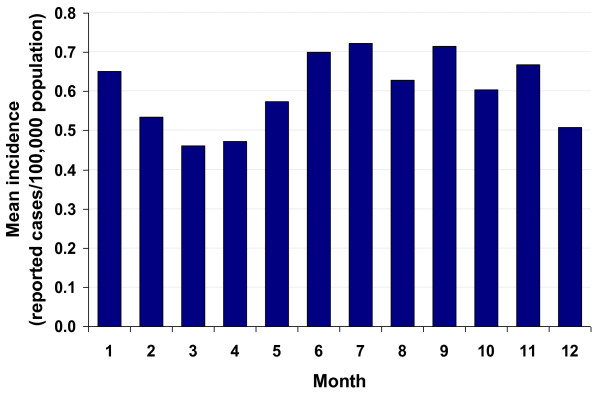
**Seasonal distribution of reported *Yersinia enterocolitica *infections in Germany, 2001-2008**. Data shows mean monthly incidence per 100,000 population.

The total number of yersiniosis cases reported in the time period 2001-2008 was 47,627. The annual number ranged from 4,354 to 7,540 (Table 1), corresponding to an annual incidence of 5.3 to 9.2 infections per 100,000 population, with an average annual incidence of 7.2 infections per 100,000 population. Of all the reported *Yersinia enterocolitica *infections, 99.3% had been laboratory-confirmed and 0.7% had been confirmed epidemiologically. Of the laboratory-confirmed infections, 92% had been culture-confirmed. A downward trend in the annual number of reported *Y. enterocolitica *cases and, correspondingly, in the incidence has occurred since 2002 across all age groups and all German federal states, with an overall decrease of 42% in reported cases from 2002 to 2008 (Table 1). In contrast to other important zoonotic enteric diseases, e.g., those caused by *Salmonella *spp. or *Campylobacter *spp., which typically peak during the summer months, the seasonal distribution of reported *Y. enterocolitica *infections was relatively uniform, with only a slight increase in June, July, and September. The lowest number of *Y. enterocolitica *infections was reported in March and April (Figure 1). The majority of infections with *Y. enterocolitica *(about 98%) was reported as single cases, i.e., with no apparent links to other cases. Over the study period, 19 to 53 clusters of yersinioses that affected a total of 40-156 persons were reported to the RKI each year. Most clusters consisted of just 2 cases, typically from the same household. Clusters with ≥5 epidemiologically related cases were reported only once or twice a year (data not shown).

**Table 1:** Annual number of reported *Yersinia enterocolitica *infections in Germany according to serotype

	Year							
	2001	2002	2003	2004	2005	2006	2007	2008
O:3	4304	5227	4826	4672	4306	3945	3935	3361
O:9	279	325	269	325	282	256	264	277
O:5,27	32	29	35	44	48	59	35	30
O:8	1	0	0	5	14	16	8	18
Other	175	342	432	185	149	155	118	145
No information on serotype	2404	1617	1015	953	829	730	628	523

Total	7195	7540	6577	6184	5628	5161	4988	4354

The total number of reported infections was 47,627. For serotypes other than O:3, O:9, O:5,27 and O:8, serotype specification was not available from the surveillance data ("other" serotypes). Not all reported infections contained information on serotype ("no information on serotype").

### Demographic distribution

**Figure 2:**
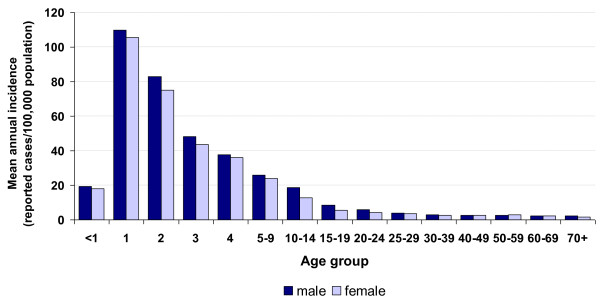
**Reported *Yersinia enterocolitica *infections in Germany by age group and sex, 2001-2008**. Data shows mean annual incidence per 100,000 population. Note that age group ranges vary.

*Y. enterocolitica *infections occurred more frequently in boys and men than in girls and women, with mean annual incidences of 8.0/100,000 population and 6.5/100,000 population, respectively. Children were more frequently affected than adults. The average annual incidence of *Y. enterocolitica *infections in children < 5 years of age was about 12-fold higher than the average in the German population aged ≥ 5 (57.6/100,000 population vs. 4.9/100,000 population, respectively). The highest incidence of reported *Y. enterocolitica *infections occurred among one-year-old children (107.9/100,000 population) (Figure 2).

### Geographical distribution

**Figure 3:**
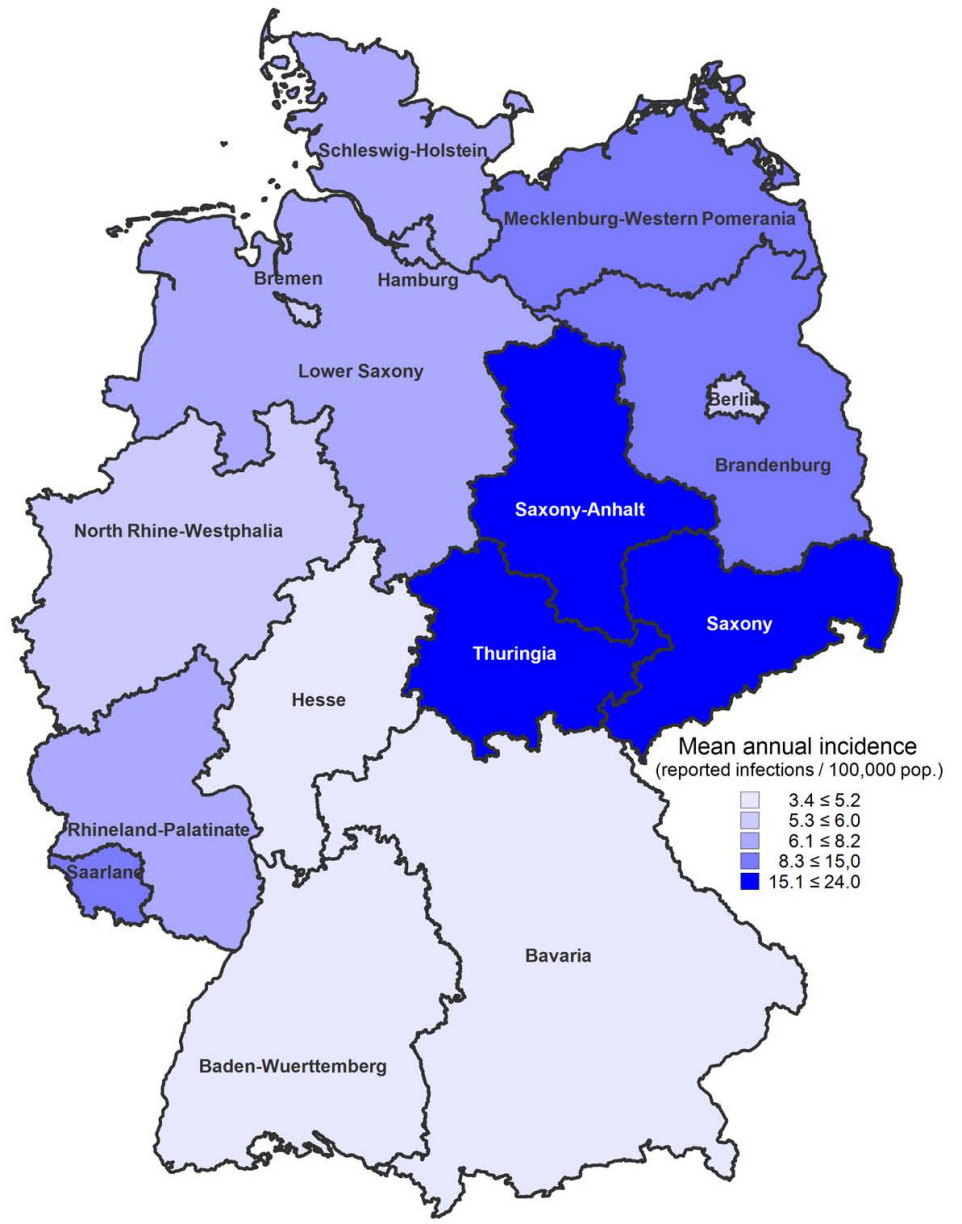
**Reported *Yersinia enterocolitica *infections in German federal states, 2001-2008**. Data on the map shows mean annual incidence per 100,000 population by federal state.

Each year, about 97% of reported yersiniosis infections with available information on the most likely country of infection were acquired domestically (Table 2). The mean annual incidence of reported *Y. enterocolitica *infections varied across the 16 German federal states, being highest in the eastern states Thuringia (23/100,000 population), Saxony (17/100,000 population), and Saxony-Anhalt (17/100,000 population), and lowest in the southern states Bavaria (5/100,000 population) and Baden-Wuerttemberg (3/100,000 population) (Figure 3). High overall annual incidences in federal states were mainly due to high incidences among children. Across federal states, mean annual incidences among children < 5 years of age differed by a factor of almost 30 (e.g., Thuringia: 413/100,000 population, Baden-Wuerttemberg: 14/100,000 population). In comparison, incidences among adults (≥18 years) differed only by a factor of about 3 across federal states (e.g., Mecklenburg-Western Pomerania: 6/100,000 population, Baden-Wuerttemberg: 2/100,000 population). Over the period of analysis, the number of reported yersinioses decreased in all federal states.

**Table 2:** Domestically acquired and imported *Yersinia enterocolitica *infections, 2001-2008

	Serotype (%)					
Most likely country of infection	O:3	O:9	O:5,27	O:8	Other	Total
Germany	97.4	96.4	95.8	95.1	96.7	97.3
Other European countries	1.9	1.9	1.5	0.0	1.9	1.9
Non-European countries	0.7	1.7	2.6	4.9	1.4	0.8

Reported frequency of country of infection is listed for different serotypes (as percentages). Total number of reported serotypes with available information on country of infection: O:3, n = 30,513; O:9, n = 2,006; O:5,27, n = 265; O:8, n = 61; other serotypes, n = 1,434. Category "other European countries" includes EU member states (without Germany) plus Iceland, Liechtenstein, Norway, and Switzerland.

### Serotypes

By far the most commonly reported human pathogenic *Y. enterocolitica *serotype was O:3 (Table 1). Of all notified cases with data on serotype, 89% were attributed to serotype O:3, 6% to serotype O:9, 0.8% to serotype O:5,27, 0.2% to serotype O:8, and 4% to other, non-specified serotypes. The number of reported cases with no available information on serotype decreased annually from about 33% in 2001 to 12% in 2008 (Table 1). The distribution of serotypes among reported cases varied according to age group. Over 90% of *Y. enterocolitica *infections among persons < 20 years of age were caused by serotype O:3, compared with 70% among patients ≥60. The proportion of infections caused by serotype O:9 was higher in adults ≥40 years of age than in younger patients (Table 3).

**Table 3:** Percentage of reported *Yersinia enterocolitica *serotypes according to age group, 2001-2008

	Serotype (%)				
Age group (years)	O:3	O:9	O:5,27	O:8	Other
0 to 4	92.7	4.1	0.3	0.1	2.9
5 to 9	93.3	3.6	0.3	0.1	2.6
10 to 19	91.3	4.6	0.5	0.1	3.5
20 to 39	84.6	7.7	1.4	0.4	5.9
40 to 59	76.3	11.2	2.0	0.3	10.2
60 and older	70.3	15.9	3.5	0.6	9.6

Total	88.8	5.8	0.8	0.2	4.4

Total number of serotypes: O:3, n = 34,576; O:9, n = 2,277; O:5,27, n = 312; O:8, n = 62; other serotypes, n = 1,701. For serotypes other than O:3, O:9, O:5,27 and O:8, serotype specification was not available from the surveillance data ("other" serotypes). Percentages may not add up to 100.0% due to rounding.

The causative agent was classified as *Y. enterocolitica *serotype O:3 in 89% of reported infections that were acquired in Germany or other countries of the EU and the European Free Trade Association (EFTA: EU plus Iceland, Liechtenstein, Norway, Switzerland). This percentage was only 77% when *Y. enterocolitica *infections were acquired in non-European countries. *Y. enterocolitica *serotype O:8 infections were more frequently imported from non-European countries than infections due to other serotypes (Table 2).

### Clinical aspects

Diarrhea and abdominal pain were the most common symptoms of reported *Y. enterocolitica *infections (Table 4). Compared to other serotypes, abdominal pain and fever were reported less frequently (34 and 11%, respectively), but diarrhea, vomiting and tenesma were reported more frequently (94, 10, and 3%, respectively) with O:8 infections. The percentage of patients with *Y. enterocolitica *infection for which a hospital stay was reported remained stable at about 17% throughout the observed time period regardless of diagnosed serotype, with the exception of serotype O:8 (with 39% of patients being hospitalized). The proportion of hospitalized patients varied with age, being highest for teenagers (10-19 years; 28% hospitalized) and elderly patients (≥60 years of age; 25% hospitalized). The median length of stay in the hospital was 4 days, but this varied with sex, age group, and serotype. It became prolonged in patients that were female, ≥20 years of age, and when isolates belonged to serotypes other than O:3. The hospitalization rate was highest (34%) and the median length of hospital stay was 8 days, when the pathogen was of serotype O:8. Between 2001 and 2008, 14 deaths due to *Y. enterocolitica *infection were reported to the national surveillance database. Six of the deceased persons were over 60 years. Serotypes of the *Y. enterocolitica *isolates were reported for 7 of the 14 deaths and included O:3 (5 deaths) and O:9 (2 deaths).

**Table 4:** Symptoms of *Yersinia enterocolitica *infections

	Serotype (%)					
Symptom	O:3	O:9	O:5,27	O:8	Other	Total
Abdominal pain	47.2	50.1	50.6	33.9	39.9	47.1
Diarrhea	85.8	82.0	77.2	93.5	73.5	85.0
Vomiting	6.1	5.1	4.5	9.7	3.9	5.9
Fever	34.0	22.9	17.9	11.3	28.5	33.0
Tenesma	0.8	0.9	2.9	3.2	1.1	0.9

Frequency of reported symptoms (as percentages) is listed for different serotypes. Total number of serotypes reported 2001-2008: O:3, n = 34,576; O:9, n = 2,277; O:5,27, n = 312; O:8, n = 62; other serotypes, n = 1,701. For serotypes other than O:3, O:9, O:5,27 and O:8, serotype specification was not available from the surveillance data ("other" serotypes). Percentages add up to > 100% because typically more than one symptom per notified case was reported.

## Discussion

German surveillance data on yersiniosis due to *Yersinia enterocolitica *infection for the time period 2001-2008 has been analyzed. The incidence of *Y. enterocolitica *infection was highest among children under 5 years of age, in particular one-year-old children. In addition to consumption of pork, other risk factors of yersiniosis need to be considered for this age group. A recent case-control study conducted in Sweden among children < 7 years of age identified, in addition to pork consumption, contact to domestic animals, in particular dogs and cats, and use of a pacifier as risk factors [8]. Age-specific risk factors, with evidence of environmental transmission, have also been demonstrated in infections with Shiga toxin-producing *E. coli *(STEC) and *Campylobacter *spp. [15,16]. A high incidence of yersiniosis among young children has been reported in other countries, and acute diarrheal illnesses caused by enteric pathogens other than *Y. enterocolitica*, e.g., infections with *Salmonella *spp., *Campylobacter *spp., or *E. coli *including STEC, also occur at high frequencies among infants [17-20]. Factors that could contribute to the high incidence in reported diarrheal illnesses in this age group may include an increased rate of exposure to enteric pathogens as a result of fecal-oral contamination [21], predisposition to infection due to an immature and unchallenged immune system [21], higher frequency of physician consultations among parents of infants [22], or higher frequency of submission of stool samples for diagnosis by physicians when infants have been affected.

Within Germany, the highest incidences of yersiniosis occurred in the federal states Thuringia, Saxony, and Saxony-Anhalt. Similar results were obtained in a joint spatial analysis of 4 gastrointestinal infectious diseases, including yersiniosis, that took underreporting into account [23]. Although drawing causal inferences from group level data to the individual level is error-prone, it is interesting to note that, according to a recent national nutrition study, consumption of meat products and sausages was relatively high in the states with the highest incidences of yersiniosis (e.g. Saxony and Thuringia). Unfortunately, published data do not specify the type of meat consumed, making it unclear whether these results are also true for pork products [24]. The number of reported *Y. enterocolitica *infections did not show seasonal variability, which may support the hypothesis that the infection is transmitted via food items that are consumed consistently throughout the year, such as meat and meat products [24], rather than being associated with unknown environmental factors. Besides, environmental transmission of zoonotic enteric pathogens, direct contact to animals, for example, seems to be more pronounced in the summer, contributing to the seasonal peak incidences associated with warmer months, as in illnesses caused by STEC and *Campylobacter *spp. [15,16].

Children were more frequently infected with *Y. enterocolitica *O:3 than adults, whereas adults ≥ 40 years of age were more frequently infected by *Y. enterocolitica *serotype O:9 than younger age groups. Prior exposure of children to *Y. enterocolitica *O:3 may conceivably provide some protection against acute infections due to the same serotype later in life, but not necessarily from other serotypes. Hospitalization was reported for 17% of patients infected with *Y. enterocolitica*, which is lower than the proportion of hospitalizations among reported *Salmonella *spp. infections in Germany (24%), but slightly higher than in reported *Campylobacter *spp. infections (14%) (unpublished data). Hospitalization was longer than the median of 4 days when serotypes other than O:3 were diagnosed. Hospitalization rate was highest and length of hospital stay was twice as long as the median when infection was due to serotype O:8, which may support the findings that the course of disease is more severe with this serotype [25]. However, the total number of reported cases infected with serotype O:8 was low (62 reported cases over the study period) and insufficient for a more detailed analysis. Interestingly, the hospitalization rate was relatively high (28%) among teenagers (10-19 years). Symptoms of yersiniosis can resemble symptoms of appendicitis in this age group (pseudoappendicitis), which may account for more frequent hospitalizations [26] and unnecessary appendectomies among teenagers [27].

In Germany, the trend in the number of reportable cases of yersiniosis has been downward since 2002. The reason for this is uncertain, but can be observed for other gastrointestinal infections caused by enteric pathogens, e.g., salmonellosis, as well [28], with the exception of *Campylobacter *spp. infections. Improved food safety control measures and better hygiene measures during food preparation at the consumer level are possible explanations for the continuing decrease of *Y. enterocolitica *infections. Compared to other European countries, the incidence of yersiniosis in Germany remains relatively high. For example, in 2007, the overall incidence was about 2-fold higher (6.1/100,000 population) than the average in all European countries reporting to the European Centre for Disease Control and Prevention (ECDC) (2.9/100,000 population) [12]. Several reasons need to be considered: First, variability in reporting systems, frequency of diagnosis, and degree of underreporting among European countries may contribute to incidence differences. Second, since consumption of pork is a risk factor of yersiniosis, food preferences will play an important role. Pork is the most frequently consumed meat in Germany with an annual consumption of about 40 kg per capita [29]. Third, prevalence and concentration of *Y. enterocolitica *in food-producing animals and products made therefrom can also result in incidence differences among EU countries.

Surveillance data have their inherent limitations. For example, routine surveillance captures only a fraction of cases occurring in the population. Thus far, the degree of under-ascertainment remains to be systematically addressed in Germany. Based on studies that were conducted in other countries, it is estimated that for each culture-confirmed case of acute diarrheal illness, between 5 and 68 undiagnosed cases occur in the community [22,30,31]. Furthermore, a more severe course of disease is more likely to precipitate medical evaluation [32,33], as is, probably, young age of the patient. Consequently, surveillance data are unlikely to be representative for the entirety of yersiniosis cases within the community. Surveillance data do not typically include detailed clinical information on every reported case, e.g., the presence of chronic diseases, cause of death, or detailed information on the laboratory diagnostic procedures. Despite these limitations, analysis of surveillance data can provide a good overview of the distribution of yersiniosis within the German population. However, identifying the risk factors of *Y. enterocolitica *infections, in particular among young children, requires analytical epidemiological methods. A case-control study is currently being conducted to elucidate and quantify the most important risk factors of *Y. enterocolitica *infections in Germany and assess sequelae-associated risk factors, with the aim of recommending effective preventive measures that will improve disease control.

## Conclusions

In Germany, yersiniosis is a zoonotic enteric disease with public health relevance because of its high incidence and the possible sequelae. Young children are affected most frequently, in particular one-year-old children, but incidence in this age group varies markedly from state to state. More research effort is required to elucidate risk factors of *Yersinia enterocolitica *infections, especially in young children.

## Competing interests

The authors declare that they have no competing interests.

## Authors' contributions

BR analyzed the data and wrote the manuscript. KS and DW critically reviewed the manuscript. All authors read and approved the final version.

## Pre-publication history

The pre-publication history for this paper can be accessed here:

http://www.biomedcentral.com/1471-2458/10/337/prepub
